# Effects of tyrosine kinase inhibitors alone or in combination with vaccine on tumor-infiltrating myeloid cells

**DOI:** 10.1186/2051-1426-1-S1-P183

**Published:** 2013-11-07

**Authors:** Renee N Donahue, Italia Grenga, Jeffrey Schlom, Benedetto Farsaci

**Affiliations:** 1 Laboratory of Tumor Immunology and Biology, CCR, NCI, NIH, Bethesda, MD, USA

## Purpose

Tyrosine kinase inhibitors (TKIs) are considered good candidates for combination with cancer immunotherapy due to their ability to decrease myeloid-derived suppressor cells (MDSCs) in peripheral blood. However, very little is known about the effects of TKIs on myeloid cells in the tumor microenvironment. In this study we evaluated the effect of combining TKIs with vaccine on the frequency and activation of myeloid cells in the tumor microenvironment.

## Methods

Flow-cytometry analysis of tumor infiltrating myeloid cells was performed in colon carcinoma (MC38-CEA^+^ in CEA-transgenic C57BL-6 mice, n=18) and breast cancer (4T1 in Balb-c mice, n=30). Mice received sunitinib or sorafenib for 21 days either alone or in combination with vaccine, which consisted of MVA-TRICOM and the tumor-associated antigen CEA in colon and TWIST in breast models. We evaluated granulocytes (Gr1^+^CD11b^-^), MDSCs (Gr1^+^CD11b^+^), and tumor-associated macrophages (TAMs, Gr1^-^CD11b^+^). Markers utilized to identify activation were FasL [1], CXCL9 [2], CD31 [3], and CD105 [4].

## Results

In the MC38-CEA^+^ model, either TKI (sunitinib or sorafenib) with or without vaccine increased the percentage of granulocytes and MDSCs and decreased TAMs, and increased the expression of 4 of 4 activation markers in MDSCs and TAMs. In contrast to the colon model, in 4T1 tumors, the frequency of granulocytes, MDSCs and TAMs were unaltered by any treatment. However, the expression of 2 of 4 activation markers (CXCL9 and CD105) increased in MDSCs and TAMs only when either TKI was combined with vaccine, and not with either TKI or vaccine alone.

## Conclusions

In one model (MC38-CEA^+^colon), addition of vaccine to either TKI (sunitinib or sorafenib) had no additional effect on the frequency or activation of tumor infiltrating myeloid cells. In another model (4T1), activation markers on tumor infiltrating myeloid cells were elevated only when TKIs were combined with vaccine. These studies provide evidence that the addition of vaccine to TKI, in some cases, enhances myeloid cell activation at the tumor.
